# Characterization of two common 5' polymorphisms in *PEX1 *and correlation to survival in PEX1 peroxisome biogenesis disorder patients

**DOI:** 10.1186/1471-2350-12-109

**Published:** 2011-08-16

**Authors:** Sven Thoms, Sabine Grønborg, Jana Rabenau, Andreas Ohlenbusch, Hendrik Rosewich, Jutta Gärtner

**Affiliations:** 1 Department of Pediatrics and Pediatric Neurology, University Medical Center, University of Göttingen, Robert Koch Str. 40, 37099 Göttingen, Germany

## Abstract

**Background:**

Mutations in PEX1 are the most common primary cause of Zellweger syndrome. In addition to exonic mutations, deletions and splice site mutations two 5' polymorphisms at c.-137 and c.-53 with a potential influence on PEX1 protein levels have been described in the 5' untranslated region (UTR) of the *PEX1 *gene.

**Methods:**

We used RACE and in silico promoter prediction analysis to study the 5' UTR of *PEX1*. We determined the distribution of *PEX1 *5' polymorphisms in a cohort of 30 Zellweger syndrome patients by standard DNA sequencing. 5' polymorphisms were analysed in relation to the two most common mutations in *PEX1 *and were incorporated into a novel genotype-phenotype analysis by correlation of three classes of *PEX1 *mutations with patient survival.

**Results:**

We provide evidence that the polymorphism 137 bp upstream of the ATG codon is not part of the UTR, rendering it a promoter polymorphism. We show that the first, but not the second most common *PEX1 *mutation arose independently of a specific upstream polymorphic constellation. By genotype-phenotype analysis we identified patients with identical exonic mutation and identical 5' polymorphisms, but strongly differing survival.

**Conclusions:**

Our study suggests that two different types of *PEX1 *5' polymorphisms have to be distinguished: a 5' UTR polymorphism at position c.-53 and a promoter polymorphism 137 bp upstream of the PEX1 start codon. Our results indicate that the exonic *PEX1 *mutation correlates with patient survival, but the two 5' polymorphisms analysed in this study do not have to be considered for diagnostic and/or prognostic purposes.

## Background

Peroxisome biogenesis disorders (PBD, MIM #601539) are a group of autosomal recessive diseases characterized by a severe developmental and metabolic phenotype [1]. PBD comprise the Zellweger syndrome (ZS) spectrum and rhizomelic chondrodysplasia punctata. The ZS spectrum consists of three overlapping clinical phenotypes representing a continuum of disorders, ranging form infantile Refsum disease (IRD, MIM #266510) as the mildest variant, to neonatal adrenoleukodystrophy (NALD, #MIM 202370) and to ZS (MIM #214100) as the prototype and most severe form with a life expectancy of less than one year. ZS patients often show characteristic dysmorphic features and severe neurodevelopmental brain abnormalities like polymicrogyria and pachygyria. Clinically patients present with muscular hypotonia and poor feeding, seizures, eye abnormalities, liver dysfunction and skeletal defects.

ZS is caused by mutations in *PEX *genes, encoding proteins (peroxins) required for the biogenesis of peroxisomes [1,2]. Peroxisomes are ubiquitous cellular organelles with functions in ether lipid biosynthesis, α-oxidation of phytanic acid, β-oxidation of very long chain fatty acids (VLCFAs) and of bile acid precursors and docosahexaenoic acid (DHA) precursors, and in hydrogen peroxide metabolism [3]. Peroxisomes are versatile organelles which can be induced and regulated according to metabolic needs [4]. In humans, 16 evolutionarily conserved peroxins are known, mutations in 13 of which are associated with disease. The peroxins PEX5 and PEX7 are cytosol-based import receptors for peroxisomal matrix proteins. The import receptor PEX5 loaded with matrix proteins can associate with most peroxisomal proteins in the cytosol and is involved in the translocation of these proteins into the peroxisome. According to one model, a downstream complex of two ATPases, PEX1 and PEX6, and the peroxisomal membrane protein PEX26 are thought to mediate recycling of PEX5 into the cytosol [5,6]. This process depends on ubiquitination of PEX5 during the translocation process at the peroxisomal membrane. Models of peroxisome protein import have been reviewed recently [7,8].

*PEX1 *and the paralogous *PEX6 *gene encode members of the AAA (ATPases associated with diverse cellular activities) family of proteins. PEX1 and PEX6 comprise two AAA domains. The *PEX1 *gene spans about 41.5 Mbp and is composed of 24 exons, encoding an mRNA of 4.4 kbp. Mutations in *PEX1 *are the most common primary cause of Zellweger syndrome and account for roughly two thirds of all PBD patients [9-14]. The most commonly found mutation in *PEX1 *is c.2528G > A leading to an exchange of Gly to Asp in the PEX1 protein [15-17]. The second most frequent mutation is the insertion 2097_2098insT leading to a frameshift and nonsense mutation after I700 [9,18]. These common *PEX1 *mutations have been included in genotype phenotype analyses showing that the missense mutation is associated with the mild end of the clinical spectrum and the insertion with more severly affected patients [1]. This correlation proves true in the majority of cases, but exceptions can be observed in which same genetic constellation leads to largely divergent phenotypes. Such cases make it necessary to look for potential effects that modulate patient outcome on the basis of a given primary defect. More recently, two polymorphisms have been described upstream the *PEX1 *coding region. One is a C-to-G transversion located 53 nucleotides upstream the *PEX1 *ATG (c.-53C > G). The other is a T-to-C transition, c.-137T > C. The c.-137 T > C polymorphism resides in a potential in-frame ATG codon that could expand the *PEX1 *ORF by 138 bp. This ATG, however, could be shown not to represent a translational start codon [19]. Reporter studies by Maxwell et al. suggest that the -137C polymorphism results in reduced expression of PEX1 whereas the -53G polymorphism increases expression of PEX1 [19]. A combination of both polymorphisms results in near-normal expression of PEX1 in a reporter experiment [19]. Polymorphisms modulating the PEX1 protein level could possibly modulate the disease phenotype of PEX1 patients. An analysis of polymorphisms and *PEX1 *mutations in 25 PEX1-deficient patients suggested that the c.-137T > C transition cosegregates with the c.-53C > G transversion. In addition, both polymorphisms were found to cosegregate with the 2097_2098insT mutation [19].

To evaluate the potential influence of known 5' polymorphisms in *PEX1 *on the disease phenotype we analysed the 5' UTR in more detail. We provide evidence that the -137C polymorphism is not a 5' UTR but rather a promoter polymorphism. We included both polymorphisms in a genotype-phenotype analysis of 30 PBD patients with *PEX1 *mutations representing the whole spectrum of clinical phenotypes.

## Methods

### Collection of patients

5'UTR polymorphisms of 30 patients with mutations in the *PEX1 *gene were analyzed. The patients had been referred to the Department of Pediatrics and Pediatric Neurology at the University of Göttingen for diagnostic evaluation of peroxisome biogenesis disorder and through the German LEUKONET. Informed consent has been obtained from guardians of all patients. The work presented in this study was conducted under the approval of the local ethics committee. The clinical phenotype and plasma biochemical abnormalities pointed towards a peroxisome biogenesis disorder and *PEX1 *gene mutations were identified by direct sequencing. *PEX1 *mutations of this patient cohort are previously published in part [12]. In some instances PEX1 deficiency was further confirmed by complementation analysis [14].

### Extraction of genomic DNA from fibroblasts and blood

Genomic DNA was extracted from 10^7 ^cells. After trypsinization, cells were washed in PBS, resuspended in 800 μl lysis buffer (50 mM Tris-HCl, pH 8.0, 100 mM EDTA, 0.5% SDS) and treated with 50 μg proteinase K for 16 hrs at 55°C. DNA was extracted by a standard protocol using phenol-chloroform. Genomic DNA from blood was extracted using the DNeasy Blood&Tissue Kit (Qiagen, Hilden, Germany) following the instructions of the manufacturer.

### RNA extraction and cDNA-Synthesis (Reverse Transcription)

RNA was extracted from fibroblasts using peqGOLD TriFast (Peqlab, Erlangen, Germany). For first strand synthesis we used the SuperScriptIII First-Strand Synthesis System (Invitrogen, Darmstadt, Germany) with 1 μg total RNA together with Oligo(dT_20_) primers according to the specification of the manufacturer.

### Determination of polymorphisms and haplotype analysis

Single nucleotide polymorphisms at c.-53 C > G und c.-137 T > C in the *PEX1 *gene, and exonic mutations were analysed by sequencing after PCR amplification using the primers listed in the table in additional file 1. PCR reactions were performed in a Thermocycler T3000 (Biometra, Göttingen, Germany) and analysed on 1% agarose gels. All PCR products were purified using the High Pure PCR Purification Kit (Roche, Grenzach-Wyhlen, Germany) following the instructions of the manufacturer. For DNA sequencing the BigDyeTerminator v3.1 Cycle Sequencing kit (Applied Biosystems, Darmstadt, Germany) was used. Sequencing reactions were purified by DNA precipitation, dissolved in 10 μl Hi-Di formamid (Applied Biosystems) and analysed on a 3100-Avant Genetic Analyzer (Applied Biosystems). Abi Prism 3100 Genetic Analyzer Data Collection Software 2.0 (Applied Biosystems) and Laser Gene Sequencing Analysis Software 5.1. (Dnastar, Madison, WI, USA) were used for sequence analysis.

### Rapid amplification of cDNA ends (RACE)

The 5'/3' *RACE *Kit (Roche) was used to characterize the transcriptional start of the *PEX1 *mRNA following the manufacturer's protocol. Briefly, *PEX1 *specific reverse primers (SP1, SP2, SP3, respectively) and Reverse Transcriptase were used to synthesize cDNA extending to the 5' end of the *PEX1 *mRNA. The 3' end of the resulting cDNA was polyadenylated using Terminal Transferase. The resulting cDNA was PCR amplified using internal *PEX1 *specific primers (SP2, Pex1ex2, SP1, respectively), and an oligo-dT anchor primer. This step was followed by a nested PCR (primers Pec10 or SP2, respectively, and the anchor primer). PCR products were directly cloned into pGEM-T-Easy Vectors I (Promega, Mannheim, Germany) and sequenced using the T7 and SP6 sites of the vector. For primer sequences see table in additional file 1.

## Results and Discussion

### Definition of the *PEX1 *5'UTR

**Figure 1:**
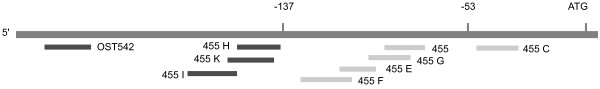
**Analysis of the 5' region of *PEX1***. Schematic representation of the *PEX1 *5' region. Positions c.-137, c.-53 and the start codon are marked. Primers that in combination with the reverse primer Pec10 or *PEX1*- Ex 2 (Exon 2) give PCR products are marked in light gray (cDNA template). Primers that do not yield PCR products are marked in dark gray. In our analysis only primers downstream of c.-137 could be used to amplify sequences from *PEX1 *cDNA. These results were confirmed by RACE.

Both 5' polymorphisms, c.-137 T > C and c.-53 C > G, have been described as 5 'UTR polymorphisms implying they become part of the mRNA. The *PEX1 *5'UTR was reported to extend up to c.-245 according to PCR amplification studies [19]. In PCRs with cDNA prepared from cultivated primary human fibroblasts, however, we could not amplify cDNA with primers upstream of or including c.-137 (primers 455H, 455K, 455I, and OST542 in Figure 1). Our analysis also included the primer used in a previous study [19], here designated OST542 (Figure 1). In contrast, all primers downstream of c.-137 (primers 455C, 455, 455G, 455E, 455F) could amplify the *PEX1 *cDNA. The most upstream functional primer extended up to c.-123 suggesting that the polymorphism at c.-137 is not necessarily part of the mRNA. Identification of the *PEX1 *transcriptional start is of importance because a transcriptional start at around c.-123 would render the upstream polymorphism at c.-137 a promotor polymorphism. For comparison, a database entry marked c.-96 as the *PEX1 *mRNA start (NCBI Reference Sequence NM_000466.2). To experimentally confirm the transcriptional start point of *PEX1*, we conducted a 5'RACE (rapid amplification of cDNA ends) experiment. The 5' region of *PEX1 *mRNA was reverse transcribed using a gene-specific internal primer, PCR amplified in two successive rounds and cloned into a pGEM-T vector for sequencing. The longest 5' extension of the cDNA that could be identified by this approach extended to c.-120, giving no further indication that the 5' UTR of *PEX1 *stretches as far as c.-245. To gain further insight into the *PEX1 *5'UTR/promotor region, we searched the 500 bp upstream of the ATG for putative transcription factor binding sites using the weight matrix-based Match tool within the TRANSFAC database [20,21]. Results are summarized in the table in additional file 2. This analysis shows that the region upstream c.-100 is covered by GC box elements that are bound by one of the classical upstream transcription factors, Sp1 [22], also arguing against a transcriptional start at c.-245.

In summary, PCR and RACE experiments, *PEX1 *cDNA database entries and a promoter analysis indicate that the *PEX1 *polymorphism at c.-137 is not necessarily a polymorphic site in the 5' UTR but could also be a polymorphism within the promoter. It is of interest that 242 bp upstream of the *PEX1 *start codon, the poorly characterized ORF C7orf64 starts, extending on the other DNA strand. Thus the *PEX1 *5' region, previously defined as UTR [19] coincides with the intergenic regions between *PEX1 *and C7orf64 and would comprise UTRs of both genes.

### 5' Polymorphisms in a PEX1 patient population

*PEX1 *upstream polymorphisms bear the potential to modulate the disease phenotype due to their influence on PEX1 expression levels. We assessed the presence or absence of the two known 5' polymorphisms at c.-137 and c.-53 in a well-characterized PBD patient collection with *PEX1 *mutations [12]. *PEX1 *mutations in this patient collection are deletions (11 out of 60 alleles), insertions (12/60), and point mutations (34/60). In three patients, only one affected allele has been identified so far (Table 1). The two most common mutations are the missense mutation c.2528 G > A in exon 15 (22 out of 60 alleles) resulting in an amino acid exchange of glycine to aspartate (p.Gly843Asp or G843D), and the insertion c.2097_2098insT in exon 13 (10/60 alleles) leading to a frameshift after isoleucin in position 700 and a premature termination codon (PTC). Altogether, 20 of the 60 alleles include PTCs, leading to potentially more severe phenotypes. Four constellations of the 5' polymorphisms were identified in the patient collective of 30 patients. We found the common constellation (c.-137 TT and c.-53 CC) in 13 (43%) of the patients and, likewise 11 (37%) heterozygous carriers (c.-137 TC and c.-53 GC). The 5' polymorphism at c.-137 alone in combination with a wild-type allele at c.-53 (c.-137 TC and c.-53 CC) appeared in three (10%) of the PEX1 patients. Another three (10%) were homozygous for both polymorphisms (c.-137 CC and c.-53 GG). The three patients with a c.-137T > C transition without the c.-53C > G transversion indicated that the c.-137T > C transition can be present in the absence of the c.-53C > G transversion. This constellation was not present in the patient cohort analysed by Maxwell and colleagues [19]. On the background of this polymorphism constellation PEX1 expression could potentially be reduced to a stronger extent than caused by the patients' *PEX1 *mutations alone. The polymorphic constellations identified in this study (TT CC, TC GG, CC GG, and TC CC) are four of a total of nine possible constellations. These four constelations can be explained by existence of only three alleles: -137C -53G, -137T -53C, and the rare -137C -53C. This would be the most parsimonious, but still speculative interpretation.

**Table 1:** Distribution of 5' polymorphisms in a cohort of PEX1 peroxisome biogenesis disorder patients

*Patient*	*1st mutation*	*Amino acids*	*Exon*	*hom/het*	*2nd mutation*	*Amino acids*	*Exon*	*hom/het*	*Survival (months)*	*c.-137 T > C*	*c.-53 C > G*
ZS1	c.2226+2T > C	r.2072_2416del p.Ala691_Lys806 delinsGlu	intr13	hom					2	TT	CC
ZS2	c.274G > C	p.Val92Leu	3	hom					23	TT	CC
ZS3	c.2083_2085del	p.695del Met	13	hom					3	TT	CC
ZS4	c.3691_3694del	p.Gln1231HisfsX3	23	hom					3	TT	CC
ZS5	c.2383C > T	p.Arg795X	14	het	c.2584-2A > G	p.Val530ArgfsX34	Intron 15	het	4	TT	CC
ZS6	c.3038G > A	p.Arg1013His	20	het	c.3287C > G	p.Ser1096X	21	het	5	TT	CC
ZS7	c.2528G > A	p.Gly843Asp	15	het	c.274-1G > C	r.274_357del p.Val92_Leu119del	Intron 2	het	> 170	TT	CC
ZS8	c.2528G > A	p.Gly843Asp	15	het	2nd mutation?				70	TT	CC
ZS9	c.2528G > A	p.Gly843Asp	15	hom					> 187	TT	CC
ZS10	c.2528G > A	p.Gly843Asp	15	hom					> 214	TT	CC
ZS11	c.2528G > A	p.Gly843Asp	15	hom						TT	CC
ZS12	c.2528G > A	p.Gly843Asp	15	hom					> 240	TT	CC
ZS13	c.2528G > A	p.Gly843Asp	15	hom					2	TT	CC
ZS14	c.724G > A	p.Val242Ile	5	het	2nd mutation?				3	TC	GC
ZS15	c.94_96del	p.Pro32del	1	het	c.2981T > C	p.Leu994Pro	19	het		TC	GC
ZS16	c.911_912del	p.Ser304fsX2	5	het	c.2387T > C	p.Leu796Pro	14	het	33	TC	GC
ZS17	c.2528G > A	p.Gly843Asp	15	hom					5	TC	GC
ZS18	c.2528G > A	p.Gly843Asp	15	hom						TC	GC
ZS19	c.2097_2098insT	p.Ile700TyrfsX42	13	het	c.1952_1960dup	p.W653_M654ins TVW	12	het	10	TC	GC
ZS20	c.2097_2098insT	p.Ile700TyrfsX42	13	het	c.2528G > A	p.Gly843Asp	15	het		TC	GC
ZS21	c.2097_2098insT	p.Ile700TyrfsX42	13	het	c.2528G > A	p.Gly843Asp	15	het	> 214	TC	GC
ZS22	c.2097_2098insT	p.Ile700TyrfsX42	13	het	c.3037C > G	p.Arg1013G	20	het	3	TC	GC
ZS23	c.2097_2098insT	p.Ile700TyrfsX42	13	het	c.3124A > C	F1042V		het	> 120	TC	GC
ZS24	c.2097_2098insT	p.Ile700TyrfsX42	13	het	c.2916delA	p.Gly973AlafsX16	18	het	3	TC	GC
ZS25	c.2528G > A	p.Gly843Asp	15	het	c.1439delT	p.Leu480TrpfsX2	7	het	18	TC	CC
ZS26	c.2528G > A	p.Gly843Asp	15	het	2nd mutation?					TC	CC
ZS27	c.2528G > A	p.Gly843Asp	15	het	c.2614C > T	p.Arg872X	16	het		TC	CC
ZS28	c.2097_2098insT	p.Ile700TyrfsX42	13	hom					11	CC	GG
ZS29	c.2097_2098insT	p.Ile700TyrfsX42	13	hom					5	CC	GG
ZS30	c.2528G > A	p.Gly843Asp	15	het	c.249insT	p.Leu84SerfsX24	2	het	3	CC	GG

The table displays the 5' polymorphisms and the previously identified exonic mutations in 30 PEX1 patients. Mutations c.2528 G > A and c.2097_2098insT are especially common. Homozygosity (hom.) is inferred from the absence of heterozygosity in the sequencing reaction and the absence of a second site mutation in PEX1. In three patients, ZS 8, ZS 14, and ZS 26, the second mutation is not identified. The 5' polymorphism genotypes were determined by direct sequencing. Four possible constellations of the 5' polymorphisms were identified in the patient collective. Patients' survival is indicated where this information was available. > before number of months indicates patients that are alive and reached the respective age at the last follow-up in 2010.

In sum, the allele frequency of the c.-137T > C polymorphism in our patient cohort was 33% (20/60), compared to 14.9% in the European population (NCBI single nucleotide polymorphism (SNP) database). Interestingly, the allele frequency of the c.-53C > G polymorphism was 28% (17/60) in our cohort, whereas in the healthy population it is reported to range between 4.4 and 7.8% (NCBI SNP database).

We next analysed the distribution of *PEX1 *5' polymorphisms in relation to the two most common *PEX1 *mutations. Table 2 shows the distribution of 5' polymorphisms in relation to c.2097_2098insT. Of eleven heterozygous patients (c.-137 TC and c.-53 GC), six were heterozygous for the insertion c.2097_2098insT. The remaining five patients did not carry the insertion. The three patients with the heterozygous polymorphism only at position c.-137 (c.-137 TC and c.-53 CC) did not carry the c.2097_2098insT insertion and both homozygous patients (c.-137 CC and c.-53 GG) were homozygous for c.2097_2098insT. None of our patients with a wild-type configuration at both polymorphism sites (c.-137 TT und c.-53 CC) carries the c.2097_2098insT insertions. We could thus confirm that the second most common *PEX1 *allele, 2097_2098insT, is coupled to the c.-137 T > C c.-53 C > G constellation and probably evolved on the basis of this allele (Table 2). The evolution of the 2097_2098insT allele on the basis of the c.-137 T > C c.-53 C > G constellation also explains the overrepresentation of the c.-53C > G polymorphism in our cohort.

**Table 2:** Distribution of *PEX1 *5' polymorphisms in relation to the common c.2097_2098insT mutation

No. of patients (this study)	No. of patients [19]	c.-137T > C polymorphism	c.-53C > G polymorphism	c.2097_2098insT genotype
13	8	TT	CC	-
**3**	**0**	**TC (↓)**	**CC**	**-**
5	1	TC (↓)	GC (↑)	-
6	15	TC (↓)	GC (↑)	het
2	1	CC (↓↓)	GG (↑↑)	hom

Het and hom refer to the heterozygous and homozygous presence of the c.2097_2098insT genotype, respectively. Our study identified a new genetic constellation, that is the potentially expression reducing 137T > C transition in the absence of the c.-53C > G transversion (marked in bold). The arrows refer to the expected effect on PEX1 expression [19].

Table 3 shows the distribution of these polymorphisms in relation to the most common *PEX1 *mutation, p.Gly843Asp. The 13 patients with the common sequence at positions c.-137 and c.-53 and the 11 patients with the two heterozygous 5' polymorphisms distributed equally in relation to the p.Gly843Asp mutation. One heterozygous p.Gly843Asp patient was found to carry the rare homozygous c.-137 CC c.-53 GG constellation. This indicated that the p.Gly843Asp allele can be found in combination with any of the four possible 5' alleles and that the p.Gly843Asp mutation and the 5' polymorphisms evolved independently of each other.

**Table 3:** *PEX1 *5' polymorphisms with respect to the most common mutation c.2528G > A (p.Gly843Asp)

No. of patients	c.-137T > C polymorphism	c.-53C > G polymorphism	c.2528G > A (p.Gly843Asp) genotype
2	TT	CC	het
5	TT	CC	hom
6	TT	CC	-

2	TC (↓)	GC (↑)	het
2	TC (↓)	GC (↑)	hom
7	TC (↓)	GC (↑)	-

3	TC (↓)	CC	het
0	TC (↓)	CC	hom
0	TC (↓)	CC	-

1	CC(↓↓)	GG (↑↑)	het
0	CC(↓↓)	GG (↑↑)	hom
2	CC(↓↓)	GG (↑↑)	-

Het and hom refer to the heterozygous and homozygous presence of the c.2528G > A genotype, respectively. The c.2528G > A allele is found in combination with all four 5' alleles. The arrows refer to the expected effect on PEX1 expression levels.

Tables 2 and 3 also indicate the expected effect of the polymorphisms on *PEX1 *gene expression levels following the analysis of Maxwell et al. [19]. It was found that c.-137 T > C polymorphism decreased the *PEX1 *gene expression by 50% (↓), whereas the c.-53 C > G polymorphism increased PEX1 expression by approximately 25% (↑) when analyzed in a luciferase reporter study [19]. A combination of both 5' polymorphisms did not lead to a significant change in *PEX1 *gene expression. To evaluate a possible effect of *PEX1 *5' polymorphisms on the severity of the patient phenotype we included the 5' polymorphisms in a genotype-phenotype analysis.

### Genotype-Phenotype-Correlation

**Figure 2:**
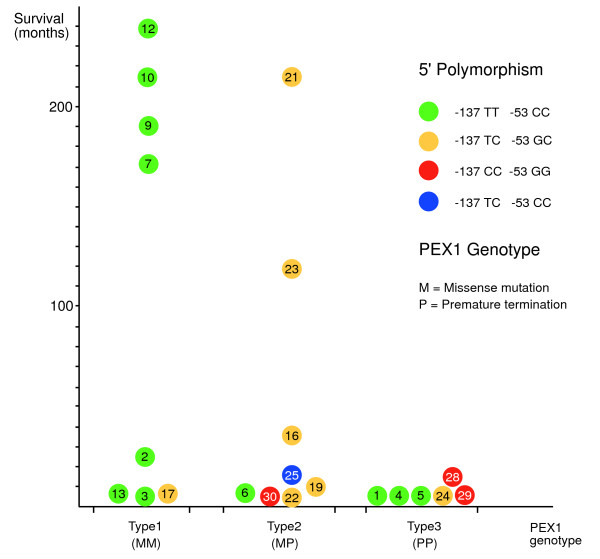
**Genotype-Phenotype-Correlation in PEX1 patients with known survival**. Patients were grouped according to their genotypes into patients with missense mutations (M) on both alleles (type 1, MM), with premature termination codons (PTCs, P) on both alleles (type 3, PP), or with a combination of a PTC with a missense mutation (type 2, MP). To limit the genotypic classes to three, we grouped short insertions that do not result in PTC together with M, and more extensive deletions together with the P alleles. 5' polymorphisms are marked in color. Numbers in circles refer to ZS patient numbers as in **Table 1**. Patients with survival of more than 100 months were alive at the time of submission of the manuscript.

With respect to the common exonic *PEX1 *mutations, a genotype-phenotype correlation has been described [9,12,23,24]. Generally, *PEX1 *alleles that lead to a PTC (P), caused by frameshift or nonsense mutations, lead to more severe phenotypes than alleles with a missense mutation (M). It is thus possible to classify patients in three groups, depending on the combinations of PTC and M: MM (type 1), PM (type 2), PP (type 3). In order to limit the genotypic classes to three, we included the patients with short insertions that do not result in PTC in the type 1 group and the more extensive deletions together with other deletions resulting in PTCs in the PTC group. We undertook this analysis with our patient cohort and plotted the time of survival as a measure of phenotypic severity on the y-axis (Figure 2).

This representation confirms that the type 3 genotype results in the most severe phenotype with an average survival of only 4.7 months (six patients). This group also includes patient ZS01 with a homozygous deletion of 115 amino acids. The average survival in the patient group with a combination of a missense mutation and a PTC (type 2) is 50.8 months (eight patients). This group also includes patient ZS19 with a three-base pair insertion in combination with the common p.Ile700TyrfsX42 mutation. Patients with missense mutations on both *PEX1 *copies (type 1) show the longest survival, 106 months on average (eight patients). The patient group generally associated with the longest survival can be divided into two groups of survival of less than 40 months or 8.2 months on average (four patients) and more than 170 months, or 203 months on average (Figure 2). It has to be noted that patients with a survival of more than 100 months were alive at the time of the submission of this manuscript, so the longer survival rates can be prognostically used as a lower limit only.

We also indicated the 5' polymorphisms within each of these groups and found that all but one patient of known survival in the type 1 group have the common 5' genotype c.-137 TT c.-53 CC. This means that the differentiation in survival in the type 1 group is not due to differences in the known 5' polymorphisms, and, more generally, that differences in 5' polymorphisms are not the cause for the different survival of patients with the same mutations in *PEX1*. On the other side, conclusions that can be drawn from this analysis are limited by the absence of a pair of patients with functionally different 5' polymorphisms and with the same exonic genotype. Extreme differences in survival of patients with identical exonic mutations and 5' polymorphisms suggest, however, that these differences cannot be attributed to the 5' polymorphisms.

## Conclusions

Our study suggests that only the *PEX1 *5' polymorphic site at c.-53 is part of the 5' UTR while the polymorphism 137 bp upstream of the PEX1 start codon might be part of the *PEX1 *promoter. The common c.2528G > A (p.Gly843Asp) mutation appears to be independent of a specific upstream polymorphism in contrast to the second most common mutation, c.2097_2098insT (p.Ile700TyrfsX42), that arose on the basis of the -137 T > C c.-53 C > G polymorphic constellation. A novel genotype-phenotype representation with only three genetic categories allows distinguishing groups of largely differing patient survival, but 5' polymorphisms do not seem to further modulate patient survival. We could not yet, however, identify patients with identical *PEX1 *mutations and functionally different 5' polymorphisms.

## Competing interests

The authors declare that they have no competing interests.

## Authors' contributions

ST, SG and JG conceived the study. ST and SG analysed the data, and wrote the manuscript. JR and AO carried out the molecular genetic studies and analyzed the data. ST and JR prepared tables and figures. ST, SG and JG supervised JR. HR provided patient genotypes and analysed the clinical data including patient survival information. All authors read and approved the final manuscript.

## Pre-publication history

The pre-publication history for this paper can be accessed here:

http://www.biomedcentral.com/1471-2350/12/109/prepub

## Supplementary Material

Additional file 1 **Primers used in this study**. Table with list of oligonucleotide primers used in this study. Click here for file

Additional file 2 **Transcription factor binding sites in the *PEX1 *5' upstream region from c.-500 to c.-1**. Table with results of an *in silico *analysis of transcription factor binding sites in the *PEX1 *5' region. Click here for file
