# Supplementary material for: Characterization of two common 5' polymorphisms in PEX1 and correlation to survival in PEX1 peroxisome biogenesis disorder patients
Source: BMC Med Genet. 2011 Aug 16;12:109. doi: 10.1186/1471-2350-12-109 (PMC3167756; doi:10.1186/1471-2350-12-109)
Supplement: Additional file 1 — Primers used in this study. Table with list of oligonucleotide primers used in this study. [file 1471-2350-12-109-S1.PDF]

**Additional file to**

# Characterization of two common 5' polymorphisms in PEX1 and correlation to survival in PEX1 peroxisome biogenesis disorder patients

Sven Thoms, Sabine Grønborg, Jana Rabenau, Andreas Ohlenbusch, Hendrik Rosewich, Jutta Gärtner

## Additional file 1.

**Primers used in this study.**

| | | |
|---|---|---|
| **Analysis of polymorphisms** | | |
| 455b | 5'CGTGCGAAGATTTGATGAACGAGGTG3' | for |
| 456b | 5'GCCCTTCTGGGTAGTCTCTGGATC3' | rev |
| **Sequencing of c.2528 (p.Gly843Asp)** | | |
| 485a | 5'CTGCTCCATCCCCAGACAACTACC3' | for |
| 488a | 5'CCGTGTGAGTCAACAAGTGTTTACTGAG3' | rev |
| **Amplification of a product including c.-137 and c.2528 (p.Gly843Asp)** | | |
| 455C | 5'GGGTCCTTTGCGGCGCTAGG3' | for |
| 455 | 5'CGATCTCCTCCGGCTCCG3' | for |
| 455G | 5'GGCCGATCGATCTCCTCCGG3' | for |
| 455E | 5'CCGCAGGGGGCGGGGCC3' | for |
| 455F | 5'AGGGGGCGGGGCCGCAG3' | for |
| 455H | 5'CCACTACAGGCTTACGGCAGG3' | for |
| 455K | 5'CCACCCACTACAGGCTTACGGC3' | for |
| 455I | 5'GACAGAAGCTTTCTTGTCCCAC3' | for |
| 455B | 5'CGTGCGAAGATTTGATGAACGAGGTG3' | for |
| Pec10 | 5'GTGGAAGGCAGGCATTTTAGTGATCAAGG3' | rev |
| OST 542 | 5'TGCGTGCGAAGATTTGATGAAC3' | for |
| PEX 1-Ex 2 | 5'CTTGGACTCTCAAATGGGGG3' | rev |
| **5'-RACE** | | |
| T7 | 5'TAATACGACTCACTATAGGG3' | for |
| SP6 | 5'ATTTAGGTGACACTATAG3' | rev |
| Oligo-dT-anchor | 5'GACCACGCGTATCGATGTCGACTTTTTTTTTTTTTTTTV3' | for |
| PCR-anchor | 5'GACCACGCGTATCGATGTCGAC3' | for |
| SP1 | 5'TCTCCCAATCATCTGCTGAGAGGG3' | rev |
| SP2 | 5'GACAAGATACCACATGGGAACATG3' | rev |
| SP3 | 5'TTGTTCAAGGGAAACAGCATGCAG3' | rev |

Oligo-dT-anchor primer contains the ambivalent nucleotide V = A, C or G.
