# Supplementary material for: Characterization of two common 5' polymorphisms in PEX1 and correlation to survival in PEX1 peroxisome biogenesis disorder patients
Source: BMC Med Genet. 2011 Aug 16;12:109. doi: 10.1186/1471-2350-12-109 (PMC3167756; doi:10.1186/1471-2350-12-109)
Supplement: Additional file 2 — Transcription factor binding sites in the PEX1 5' upstream region from c.-500 to c.-1. Table with results of an in silico analysis of transcription factor binding sites in the PEX1 5' region. [file 1471-2350-12-109-S2.PDF]

Additional file to

Characterization of two common 5' polymorphisms in PEX1 and correlation to survival in PEX1 peroxisome biogenesis disorder patients

Sven Thoms, Sabine Grønborg, Jana Rabenau, Andreas Ohlenbusch, Hendrik Rosewich, Jutta Gärtner

Additional file 2.

Transcription factor binding sites in the *PEX1* 5' upstream region from c.-500 to c.-1.

Factor name	Matrix identifier	Pos. (c.)	Strand	Core match	Matrix match	Sequence	Tissue
Tax/CREB	V$TAXCREB_02	-445	(+)	0.800	0.820	gTGTCGcataccgcc	r l
YY1	V$YY1_02	-387	(+)	1.000	0.948	tcgacGCCATcttgcctact	l
YY1	V$YY1_Q6	-382	(+)	1.000	1.000	GCCATcttg	l
PPARg:RXRa, PPARg	V$PPARG_02	-263	(+)	0.832	0.690	gcgtcGGCCAtcgtttcctgcgt	r
AhR, Arnt, HIF-1	V$AHRHIF_Q6	-245	(+)	1.000	0.999	tGCGTGcga	r l
KROX	V$KROX_Q6	-126	(-)	1.000	0.982	ggagaGGGGGcggg	r
Sp1	V$SP1_Q6	-122	(+)	1.000	1.000	agggGGCGGggcc	a r l
Sp1	V$SP1_Q4_01	-122	(+)	1.000	1.000	agggGGCGGggcc	a r l
Sp1	V$SP1_Q2_01	-121	(-)	1.000	1.000	gggGGCGGgg	a r l
Sp1	V$SP1_01	-120	(+)	1.000	0.971	ggGGCGGggc	a r l
Sp1	V$SP1_Q6_01	-120	(+)	1.000	1.000	ggGGCGGggc	a r l
Sp1	V$SP1_Q6	-107	(+)	1.000	1.000	agggGGCGGggcc	a r l
Sp1	V$SP1_Q4_01	-107	(+)	1.000	1.000	agggGGCGGggcc	a r l
Sp1	V$SP1_Q2_01	-106	(-)	1.000	1.000	gggGGCGGgg	a r l
Sp1	V$SP1_01	-105	(+)	1.000	0.971	ggGGCGGggc	a r l
Sp1	V$SP1_Q6_01	-105	(+)	1.000	1.000	ggGGCGGggc	a r l
AP-2	V$AP2_Q3	-70	(-)	1.000	0.945	tcctcgGCCTGccggg	a

Tissue specificity, a, adipose tissue; r, redox-specific; l, liver specific.
